# Supplementary material for: Tracking the Pathways of West Nile Virus: Phylogenetic and Phylogeographic Analysis of a 2024 Isolate from Portugal
Source: Microorganisms. 2025 Mar 4;13(3):585. doi: 10.3390/microorganisms13030585 (PMC11945232; doi:10.3390/microorganisms13030585)
Supplement: Supplementary file 1 [file microorganisms-13-00585-s001.zip › microorganisms-3479426-supplementary.pdf]

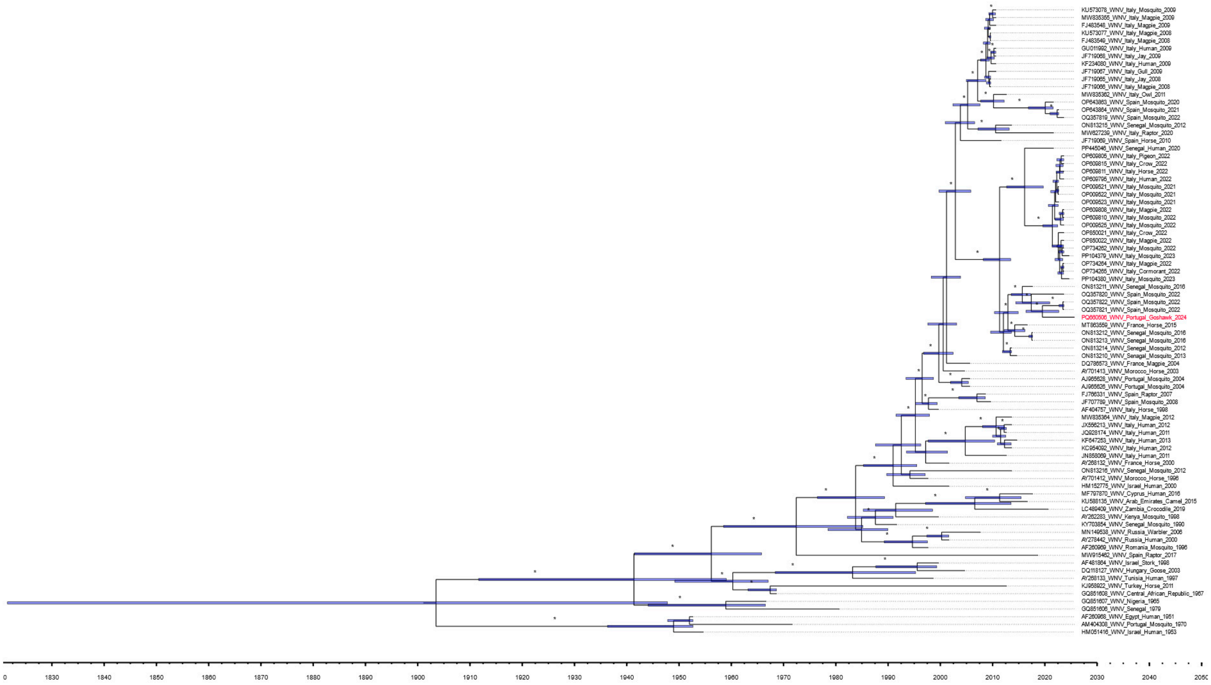

Figure S1. Time-scaled Bayesian phylogenetic tree of West Nile virus lineage 1a strains reconstructed using a constant demographic prior. Posterior probabilities at specific branch nodes that are equal or greater than 0.90 are indicated by an asterisk (*). The 95% highest posterior densities (HPDs) of the median node ages are represented by blue bars. The time of the most recent common ancestor (tMRCA) was estimated to be around 1903. Viral sequences are identified by their accession number, country of origin, host, and year of collection. The WNV/18665/PT2024 genomic sequence is indicated in red.

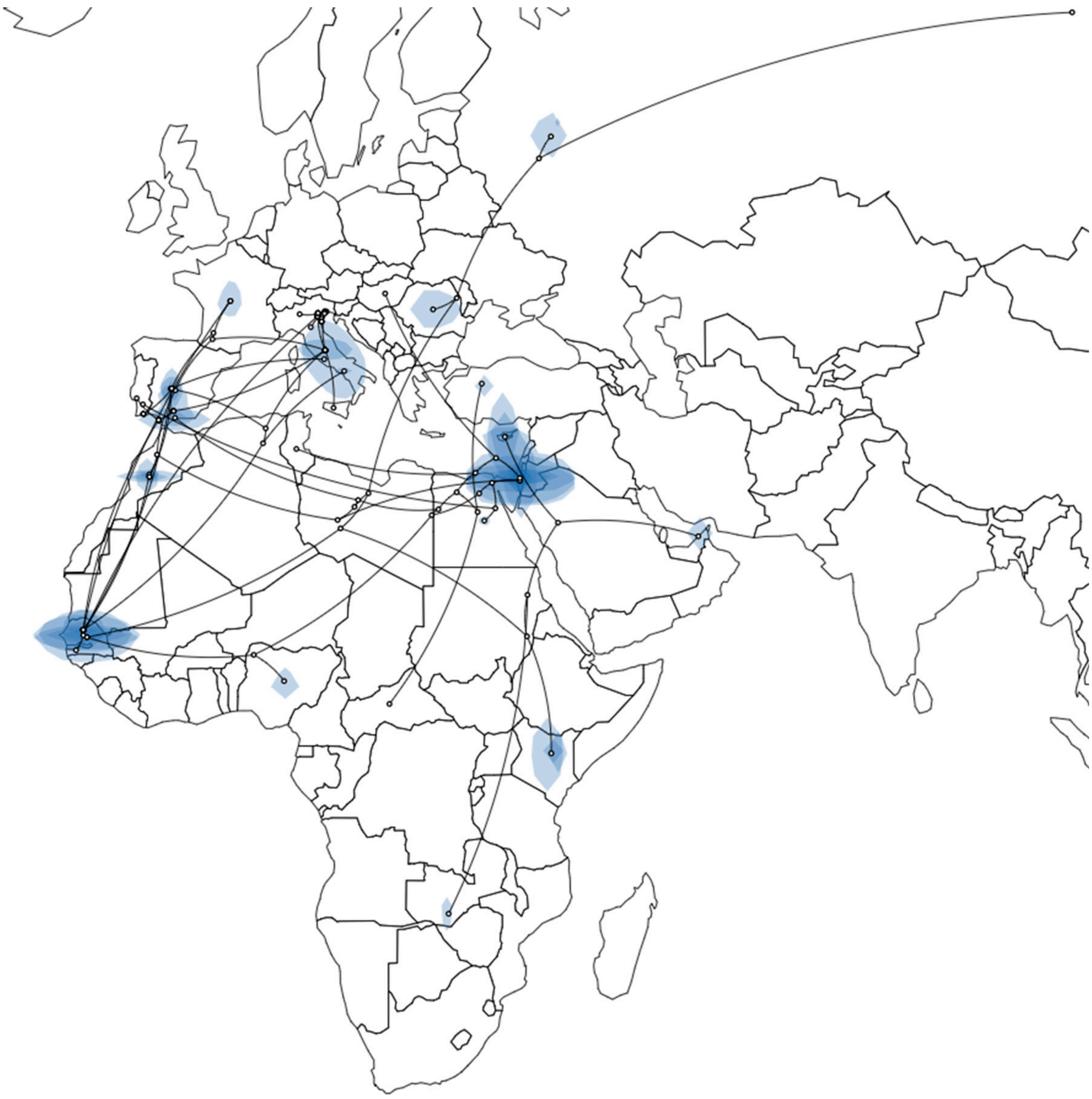

Figure S2. Phylogeographic reconstruction and diffusion pattern of the 82 L1a genomes and their ancestors based on the constant demographic prior analysis. The blue polygon areas correspond to the probability density associated with those locations.

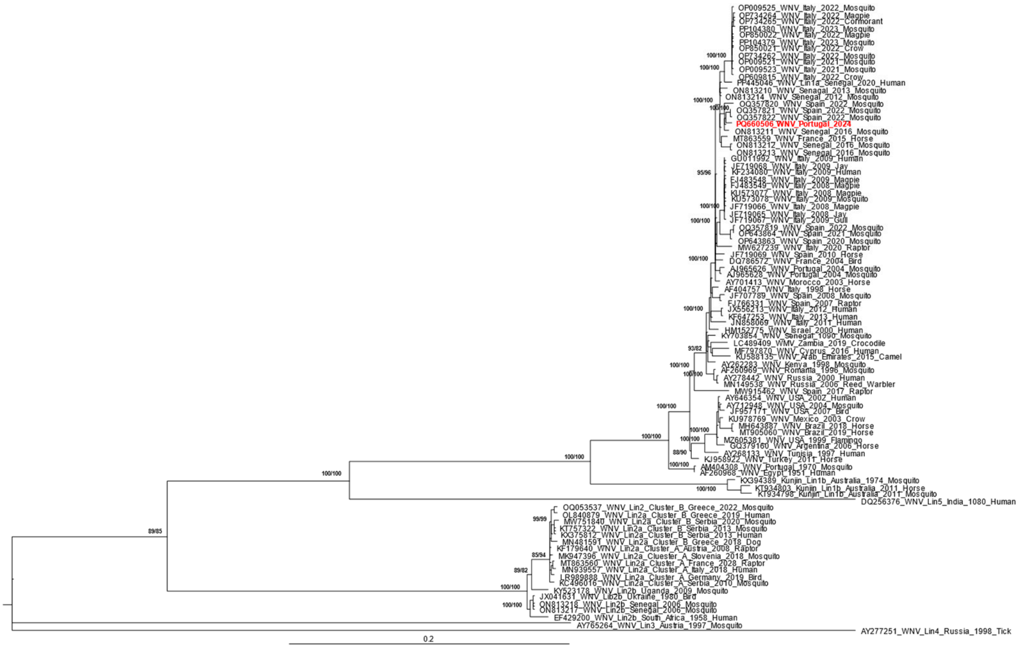

Figure S3. ML tree based on the nucleotide sequences of the coding region (ORF) of West Nile virus strains. At the main branches of the tree, the x/y bold-faced values indicated refer to their respective branch topological support, as revealed by aLRT (x) and bootstrap (y) analyses. Only values > 80% (of 1000 data resamplings), are indicated. The different WNV strains are indicated in the tree by their accession number, lineage (+ cluster type in the case of Lineage 2 viruses), geographic origin, date of sampling and host. The WNV sequence obtained in the course of this work is indicated in red.
